# Testing Motivational Appeals to Promote Legume-Enriched Foods

**DOI:** 10.3390/nu18040552

**Published:** 2026-02-07

**Authors:** Marco Gaetani, Valentina Carfora, Laura Picciafoco, Patrizia Catellani

**Affiliations:** Department of Psychology, Catholic University of the Sacred Heart, 20123 Milan, Italy; marcogaetani@outlook.it (M.G.); laura.picciafoco@unicatt.it (L.P.); patrizia.catellani@unicatt.it (P.C.)

**Keywords:** legume-enriched food, plant-based food, tailored communication, recommendation messages, food choice motives

## Abstract

**Background/Objectives:** Legume-enriched foods are conventional products reformulated with the addition of legumes and, as such, represent a sustainable alternative to animal proteins. This study investigated the effectiveness of messages based on different food choice motives to encourage search, consumption, and future intention to consume these foods. **Methods:** The study involved a representative sample of 1361 Italian adults randomly assigned to one of seven experimental conditions (i.e., health, price, sensory appeal, natural content, convenience, sustainability, mood) or a control condition. Participants received three prefactual gain messages over one week. A moderated serial mediation model was estimated to test whether the effects of message exposure on future intention to consume were mediated by product search and consumption, and whether these effects varied according to participants’ baseline intention to replace animal food with plant-based alternatives (i.e., intenders vs. non-intenders). **Results:** Reading messages focusing on mood (*B* = 0.337, *p* = 0.021), sustainability (*B* = 0.441, *p* = 0.002), health (*B* = 0.333, *p* = 0.029), and convenience (*B* = 0.364, *p* = 0.017) were associated with increased intention to consume legume-enriched foods. However, only reading sustainability messages showed a positive serial indirect effect on intention via search and consumption (*B* = 0.036, *p* = 0.044), while reading mood messages was associated with increased intention via search only (*B* = 0.243, *p* = 0.048). Among non-intenders, reading mood and health messages were associated with increased intention only when they first stimulated search behavior. Conversely, among intenders, only reading sustainability messages was associated with increased consumption. **Conclusions:** These results demonstrate the persuasive power of sustainability appeals in promoting legume-enriched food consumption and support the effectiveness of using recommendation messages tailored to the recipient’s stage of change in terms of replacing animal food with plant-based alternatives.

## 1. Introduction

Diets rich in animal products pose environmental and health challenges [1], yet global meat consumption continues to rise. Legumes, characterized by a balanced amino acid profile and versatile functional properties [2], offer a sustainable alternative, requiring far fewer resources and emitting over 90% less greenhouse gases than beef [3,4]. Despite these advantages, their consumption remains low in Western countries due to sensory and practical barriers [5,6]. Integrating legumes into food formulations may help overcome these obstacles and foster greater acceptance [7].

Legume-enriched foods, such as bread, cookies, chips, and meat analogues, represent an emerging category of novel foods (i.e., foods that were not consumed to any significant extent in the EU before May 1997 [8,9]). Promoting their consumption is increasingly relevant for both health and sustainability [5,10]. Communication strategies offer a promising tool to encourage behaviour change and the substitution of animal foods with legume-enriched alternatives [11]; however, research on persuasive communication in this area remains limited e.g., [12,13].

Building on this gap, this study investigated the effectiveness of recommendation messages emphasizing different food choice motives to promote product search, consumption, and intention to consume legume-enriched foods among a representative Italian sample. The study further examined whether product search and consumption mediated the message effect on future intention, and whether baseline motivation to reduce animal product intake moderated message effectiveness.

## 2. Theoretical Background

### 2.1. A Process-Oriented Framework for Sustainable Food Choice

Sustainable food choice is increasingly conceptualized as a process rather than a single act, in which consumers move from exploratory behaviors, such as information search, to experiential engagement and subsequent evaluation of future intentions [14].

In the context of sustainable and novel foods, this process is shaped by a dynamic interplay between motivational factors, contextual constraints, and individuals’ readiness to change. In line with the social psychology of eating, food choice motives may therefore operate as context-dependent cues, becoming more or less salient at different moments of the decision process rather than exerting uniform effects across stages [15,16].

Consistent with this perspective, the present study conceptualizes product search as an early exploratory outcome, consumption as an experiential trial behavior, and future intention as a post-experience evaluative outcome reflecting the consolidation or revision of motivational commitment. This process-oriented framework allows for the examination of how different motivational framings relate to interconnected phases of consumer engagement with legume-enriched foods, without assuming a single, stage-invariant persuasive effect.

### 2.2. Recommendation Messages to Support the Transition to a Sustainable Diet

Within the process-oriented framework outlined above, persuasive communication represents a central tool for activating motivational cues that may differentially influence consumer engagement across stages of sustainable food choice. Indeed, persuasive communication has long been shown to influence attitudes, intentions, and behaviours. When applied to sustainable food promotion, such communication can target the underlying motives that drive consumers’ food choices. A food motive refers to the specific reason for choosing certain foods in a given context [17,18], and can be classified as primary (biologically driven) or secondary (socially and culturally shaped) [19]. The Food Choice Questionnaire (FCQ) is the most widely used framework for categorizing secondary motives, identifying nine dimensions: health, mood, convenience, sensory appeal, natural content, price, weight control, familiarity, and ethical concern [18]. Cross-cultural studies have confirmed its generalizability across contexts [20,21] (see also [22] for a comparative ranking of motives across nine European countries).

Recent research on sustainable food choices has highlighted additional sustainability-related motives, such as animal welfare, environmental impact, and ethical concern, as predictors of plant-based and legume consumption [23]. In the context of alternative protein, key product-related drivers include health, taste, appearance, convenience, environmental benefits, naturalness, and price [24]. Similarly, health, price, and taste are central to the acceptance of meat products enriched with plant-based ingredients [25]. Emotional motives also play a role, with anticipated mood improvement predicting alternative protein consumption beyond traditional food motives [26].

Building on these insights, recent interventions have used messages tailored to specific food motives to promote sustainable choices e.g., [27,28,29,30]. Nevertheless, evidence is mixed: combined health and environmental claims increased willingness to pay for legume-enriched foods [12,31], whereas single-focus messages on health or environment alone did not significantly affect preferences or willingness to pay for plant-based sausages [13].

Nevertheless, no study has simultaneously compared the persuasiveness of multiple motives beyond health and sustainability in promoting novel foods. Additionally, although several behaviour change models emphasize the importance of distinguishing between different stages of adoption, such as information seeking, actual behaviour, and future intention [32,33]. Research on sustainable food communication has rarely examined all three within a single design. The mechanisms through which such motivational messages influence future consumption intention, particularly through product search and consumption, also remain unexplored.

To address these gaps, the present study formulated the following research question:

Research Question 1 (RQ1)—Does exposure to messages grounded in different food choice motives (i.e., health, price, sensory appeal, natural content, convenience, sustainability, and mood) influence recipients’ search (RQ1a), consumption (RQ1b), and future intention to consume legume-enriched foods (RQ1c)?

Are the message effects on future intention mediated by product search and consumption, either independently or sequentially (RQ1d)?

### 2.3. The Moderating Role of Consumers’ Intention to Replace Animal Foods with Plant-Based Alternatives

The intention to change dietary habits represents a key individual factor influencing acceptance of novel foods. Research consistently shows that individuals intending to change their diet differ from those who do not, particularly in the motivations and beliefs underlying their willingness to try novel foods e.g., [34,35]. For instance, individuals motivated to reduce meat consumption are generally more open to alternative foods, including legumes and legume-based products e.g., [36]. Individuals considering or already reducing meat consumption showed greater acceptance of blended meat products (i.e., traditional dishes where part of the meat is replaced by plant-based ingredients) and a stronger preference for plant-based or lab-grown alternatives over conventional meat e.g., [25,31].

Evidence also suggests that baseline intentions moderate responses to messages promoting dietary changes e.g., [37,38]. People actively trying to reduce their meat consumption tended to be more responsive to sustainability labels [37] and to messages promoting the replacement of meat with plant-based alternatives [39]. Overall, individuals already engaged in or considering a shift toward sustainable eating (“intenders”) appear more receptive to persuasive messages than those not yet contemplating such changes (“non-intenders”) [35,40]. To investigate this potential moderation effect, the following research question was formulated:

Research Question 2 (RQ2)—Do the effects of the messages on search (RQ2a), consumption (RQ2b), and future intention to consume legume-enriched foods (RQ2c) differ between intenders and non-intenders?

**Figure 1:**
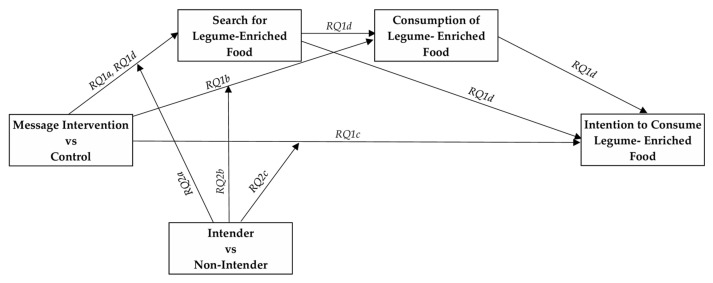
Conceptual Model Tested in the Study. Note: Message conditions were modeled as independent variables. Product search and product consumption were specified as sequential mediators of the effects of message exposure on future intention to consume legume-enriched foods (final outcome) (RQ1). Baseline intention to replace animal foods with plant-based alternatives (intender vs. non-intender) was specified as a moderator of the paths from message exposure to the mediators and the final outcome (RQ2).

Figure 1 provides a graphical representation of the tested conceptual model.

## 3. Materials and Methods

The Methods section is structured as follows. First, the study procedure and sample are described. Next, the experimental messages and measures are outlined. Finally, the data analysis strategy is presented.

### 3.1. Procedure

This randomized controlled experiment with parallel groups was conducted according to the guidelines of the Declaration and was approved by the Ethics Committee of the Department of Psychology of the Catholic University of the Sacred Heart (protocol number: 97-23; date of approval: 15 November 2023). Given the non-invasive, informational nature of the intervention, no physical harm was expected, and no psychological risks beyond minimal exposure to persuasive messages were anticipated.

Between December 2024 and January 2025, a sample of adults, representative of the Italian population in terms of gender, age, education, and region, was recruited through the SWG panel (https://www.swg.it) to participate in a study on eating habits. Eligible participants were native Italian speakers aged 18 or older. Participants were randomly allocated by the survey panel provider (SWG) to one of the seven experimental conditions or the control condition using a fully automated allocation procedure with a 1:1 allocation ratio across groups. The research team had no direct involvement in the randomisation process, and no additional restrictions (e.g., blocking or stratification) were applied.

The study consisted of a baseline assessment (T1), a one-week message exposure phase, and a follow-up assessment (T2). After providing informed consent and completing a baseline questionnaire on eating habits, participants were randomly assigned to one of seven experimental conditions or a control (see Section 3.3 “Message Intervention”). Due to the nature of the intervention, which required participants to actively read the messages, blinding of participants to the intervention was not possible. However, participants were not informed that different experimental conditions existed, nor that the message they read represented a specific experimental condition. Outcome assessment relied on self-reported measures, and data analysis was conducted after completion of data collection. Over one week, participants in the experimental groups received one message per day on days 2–4 (three messages in total), while control participants received neutral reminders. On day 7, all participants completed the post-intervention questionnaire assessing outcome variables.

### 3.2. Participants

**Figure A1:**
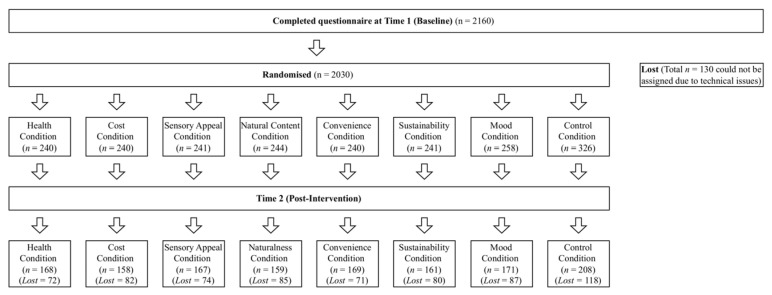
Flow Chart of Participants’ Recruitment and Drop-Out Across Conditions.

An initial sample of 2160 eligible participants completed the baseline questionnaire. After excluding 799 individuals due to technical issues (*n* = 130), incomplete follow-up (*n* = 170), failing attention checks (*n* = 387), unread messages (*n* = 36), or delayed completion (>10 days; *n* = 76), the final sample comprised 1361 participants. As shown in Figure A1, participants were evenly distributed across experimental conditions, and comparisons with the initial sample (Table A1) confirmed representativeness by gender, age, education and region was maintained. Participants’ mean age was 52.8 years (SD = 14.7, range = 18–75). 58% were women and 0.1% were non-binary or other. Overall, 37.1% had a higher education degree and 56.1% were employed. Geographically, 30% resided in north-western Italy, 19.9% in north-eastern Italy, 21.4% in central Italy, 19.5% in the south, and 9.3% on the islands (Table A2).

### 3.3. Message Intervention

Participants were randomly assigned to one of seven experimental conditions (health, price, sensory appeal, natural content, convenience, sustainability, and mood) or a control. Each condition was designed to isolate the persuasive effect of a single food choice motive, allowing direct comparison across motivational frames. Each message (20–30 words) was visually engaging and followed a standardized prefactual gain-framed structure (i.e., “If… then…”; [41]) describing the positive outcomes of consuming legume-enriched foods. This format, previously shown to be particularly effective in promoting acceptance of novel plant-based foods e.g., [36,42,43,44], allowed the persuasive effect of each motive to be examined in isolation.

In all experimental conditions, the core motivational content of each message was visually emphasized using bold text. This design choice was intended to increase the salience of the defining motivational cue and to ensure that it was clearly perceived and cognitively processed by participants. The use of typographic emphasis as an attention-guiding cue is consistent with prior evidence showing that boldface enhances visual salience, captures attention, and facilitates the processing of emphasized information e.g., [45,46].

**Figure 2:**
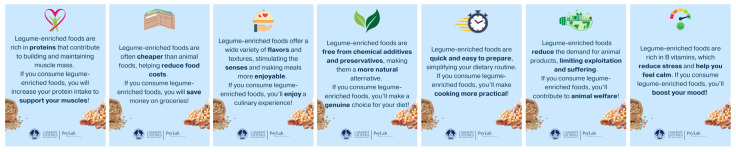
Examples of Experimental Messages by Conditions.

Figure 2 provides examples of the messages for each experimental condition, while the full list of recommendation messages can be found on OSF.

### 3.4. Measures

Two questionnaires were administered, one at baseline (Time 1—T1) and one at follow-up (Time 2—T2), both available on OSF. Table 1 summarizes the measures and their internal consistency.

**Figure A2:**
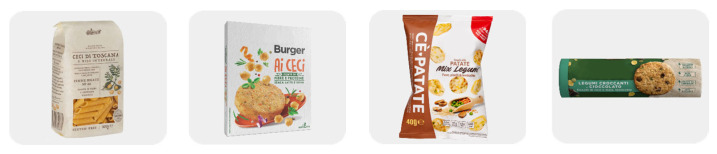
Examples of Pictures of Legume-Enriched Foods Shown to Participants Before Answering Related Questions.

At T1, before answering the questions on legume-enriched foods, participants were shown the following description: “Legume-enriched foods are common products (such as pasta, snacks, burgers, and drinks) that have been introduced to the market in a new version through the addition of legumes (such as lentils, peas, chickpeas, beans, or fava beans). The addition of legumes aims to increase the plant-based protein content of these foods, making them a viable alternative to the consumption of animal proteins (e.g., from meat or fish). Below you can see some examples of legume-enriched foods”. Participants were then shown pictures of these foods (Figure A2). Subsequently, they reported their awareness, past consumption, and intention to replace animal foods with plant-based alternatives (adapted from [47]). Participants indicating no intention to change their diet (response option = 1) were coded as non-intenders, whereas those reporting any degree of intention or having already initiated dietary change (response options = 2–8) were coded as intenders. This categorization was adopted to distinguish individuals who did not perceive any reason or motivation to reduce their consumption of animal-based foods from those who reported at least an emerging intention to do so, in line with the distinction commonly made in the literature between non-intenders and intenders e.g., [35,48]. Socio-demographic and dietary variables were also collected.

At T2, participants reported their intention to consume legume-enriched foods (adapted from [39]) and indicated whether they had searched for or consumed such products during the previous week. Search and consumption were assessed using single-item measures, as both outcomes were defined as concrete and observable behaviors rather than latent constructs [49]. Although responses were initially collected on five-point ordinal scales, distributions showed strong skewness, with minimal endorsement of higher categories, particularly for consumption (*Search*: response option 4 = 8.5%, response option 5 = 2.2%; *Consumption*: response option 4 = 5.4%, response option 5 = 0.8%). Given this distributional pattern and the short interval between message exposure and assessment, which left participants limited time for extensive search or repeated consumption, both variables were dichotomized to indicate behavioral occurrence within the observation window. Specifically, search behavior was coded as no or minimal (1–2) versus at least moderate engagement (3–5), and consumption as absent (1) versus present (2–5). Finally, message-related perceptions, including tone, involvement, trust, systematic processing, perceived freedom threat, and elicited emotions, were assessed [50,51].

### 3.5. Data Analysis

Statistical analyses were performed using Stata 17 [52] and the lavaan package 0.6.19 [53] in R 4.4.2 [54]. Composite scores were computed for constructs with at least three items. Descriptive statistics and bivariate correlations (Table 2, Table A2 and Table A3) were examined and data met acceptable thresholds for normality (|skewness| < 2, |kurtosis| < 7) [55]. Randomization and manipulation checks were performed through one-way ANOVAs and chi-square tests; dropout predictors were tested via logistic regression. Differences in message reading and evaluation across conditions were assessed using one-way ANOVAs with Bonferroni or Games–Howell post hoc tests, depending on variance homogeneity. Multicollinearity was ruled out (*r* < 0.90; VIF < 10) [56,57]. No data were missing due to forced responses.

To answer RQ1, a serial mediation model was estimated within a path analysis with seven dummy-coded conditions (control as reference) entered as exogenous variables, product search and consumption as binary sequential mediators, and intention as the final continuous outcome. Given the binary nature of the mediators, the model was estimated using diagonally weighted least squares (WLSMV) [53], with indirect and total effects specified as user-defined parameters. Model fit indices were not reported due to model saturation [58]. Specifically, the direct effects of the interventions on search and the total effects of the interventions on consumption and intention were examined to answer RQ1a–c, while the indirect effects were examined to address RQ1d.

To answer RQ2, the serial mediation model was extended to include the main and interaction effects of a dummy variable indicating whether the participant was an intender (vs. non-intender). These terms were added to the paths from the seven exogenous variables to the three endogenous variables. Conditional effects of the interventions were estimated separately for intenders and non-intenders, and if at least one was statistically significant (*p* < 0.05), Wald tests were conducted to assess whether the difference in the effects between groups was statistically significant. Only differences supported by a statistically significant Wald test were discussed in Section 4.4.

## 4. Results

### 4.1. Preliminary Analyses

Descriptive statistics (Table 2 and Table A2) indicate that 57.6% of participants intended to reduce their consumption of animal-based foods, and 78.6% were aware of legume-enriched products. Nonetheless, consumption was limited: 41.1% reported no consumption in the past month and 36.6% consumed them less than once per week. Randomization successfully balanced the conditions on all baseline variables (*p* > 0.05).

Compared to intenders, non-intenders were more likely to be men (51.1% vs. 35.2%; *χ*^2^(2) = 35.12, *p* < 0.001), older (*M* = 54.2, *SD* = 13.7 vs. *M* = 51.6, *SD* = 15.2; *t*(1359) = 3.28, *p* = 0.001), less educated (68.5% vs. 31.5%; *χ*^2^(1) = 13.28, *p* < 0.001), employed (58.9% vs. 53.9%; *χ*^2^(4) = 12.19, *p* = 0.012), omnivorous (94.1% vs. 73.9%; *χ*^2^(5) = 114.92, *p* < 0.001), unaware of legume-enriched foods (29.3% vs. 15.5%; *χ*^2^(1) = 37.26, *p* < 0.001), and to have never consumed them (61.0% vs. 26.5%; *χ*^2^(4) = 77.74, *p* < 0.001).

Messages were positively evaluated, with high mean scores for involvement (*M* = 5.02, *SD* = 1.26), trust (*M* = 4.60, *SD* = 1.13), processing (*M* = 4.75, *SD* = 1.07), positive emotions (*M* = 3.08, SD = 0.85), low perceived threat (*M* = 2.45, *SD* = 1.39) and low negative emotions (*M* = 1.20, *SD* = 0.44). Post-intervention, participants reported a high intention to consume legume-enriched foods (*M* = 4.65, *SD* = 1.45), with 36.3% searching for and 62.7% consuming such products during the study week.

Logistic regression on retention (Figure A1) showed that being man (*B* = −0.25, *S.E*. = 0.10, *p* = 0.01, *Exp*(*B*) = 0.77), younger age (i.e., aged 18–44 years; *B* = −0.56, *S.E*. = 0.11, *p* < 0.001, *Exp*(*B*) = 0.56), residing in southern Italy or the islands (*B* = −0.42, *S.E*. = 0.10, *p* < 0.001, *Exp*(*B*) = 0.65), and prior consumption (*B* = −0.21, *S.E.* = 0.06, *p* = 0.001, *Exp*(*B*) = 0.80) were associated with lower odds of completing the study. Conversely, higher education (*B* = 0.29, *S.E.* = 0.11, *p* = 0.01, *Exp*(*B*) = 1.33), following an omnivorous diet (*B* = 0.60, *S.E*. = 0.12, *p* < 0.001, *Exp*(*B*) = 1.83), and prior awareness of legume-enriched foods (*B* = 0.81, *S.E*. = 0.11, *p* < 0.001, *Exp*(*B*) = 2.26) were associated with higher odds of retention. Experimental conditions, employment, and intention status did not predict dropout.

### 4.2. Reading and Evaluating Messages

Among participants in the experimental conditions, 58% reported always reading the messages, 16.3% often, and 25.5% occasionally, with no differences across conditions (*χ*^2^(12) = 16.92, *p* = 0.15). A manipulation check confirmed accurate recognition of message content (*χ*^2^(36) = 3047.27, *p* < 0.001).

No differences emerged across conditions in perceived threat to freedom (*F*(6, 507.50) = 1.60, *p* = 0.14). Although positive emotions varied overall (*F*(6, 1146) = 2.26, *p* = 0.04), no pairwise contrasts reached significance. Significant effects were found for perceived tone (*F*(6, 1146) = 3.09, *p* = 0.01), with mood messages evaluated more positively than price and natural content messages; for message involvement (*F*(6, 1146) = 3.84, *p* < 0.001), greater for sustainability than sensory appeal and natural content messages; and for trust (*F*(6, 507.72) = 3.66, *p* = 0.001), higher for health and sustainability than price and natural content messages. Systematic processing also differed (*F*(6, 1146) = 2.35, *p* = 0.03), being stronger for sustainability than sensory appeal messages. Finally, negative emotions differed across conditions (*χ*^2^(6) = 12.74, *p* = 0.05), with mood eliciting fewer negative emotions than health and sustainability messages.

### 4.3. Serial Mediation Model

Results of the serial mediation model are shown in the first column (“All”) of Table 3 and Table A4. Notably, Table 3 shows direct effects on the three main outcomes, while Table A4 summarizes the indirect and total effects.

Examination of the direct associations between the endogenous variables revealed that, in general (i.e., among participants in the control group), searching for legume-based foods was associated with a higher likelihood of having consumed them (*B* = 0.798, *S.E*. = 0.024, *p* < 0.001). In addition, both search (*B* = 0.933, *S.E.* = 0.049, *p* < 0.001) and consumption (*B* = 0.143, *S.E.* = 0.033, *p* < 0.001) were positively associated with future intention to consume these foods, although the effect of search was substantially stronger than that of consumption in terms of magnitude.

Reading mood messages was associated with higher search (*B* = 0.261, *S.E.* = 0.132, *p* = 0.049) and intention to consume these products (*B* = 0.337, *S.E.* = 0.146, *p* = 0.021) compared to the control group, but not with consumption. A positive and statistically significant indirect effect of mood messages on intention through search was found (*B* = 0.243, *S.E*. = 0.123, *p* = 0.048), while the indirect effects through consumption and the full serial path were not significant.

Reading sustainability messages was associated with higher search (*B* = 0.311, *S.E*. = 0.134, *p* = 0.021) and intention to consume legume-based foods (*B* = 0.441, *S.E.* = 0.146, *p* = 0.002) compared to the control group, but not with consumption. Significant positive indirect effects of sustainability messages on intention were found via search (*B* = 0.290, *S.E.* = 0.125, *p* = 0.020) and through the full serial path (*B* = 0.036, *S.E.* = 0.018, *p* = 0.044), but not via consumption alone.

Reading convenience and health messages was associated with a higher intention to consume legume-based products compared to the control group (*Convenience*: *B* = 0.364, *S.E.* = 0.152, *p* = 0.017; *Health*: *B* = 0.333, *S.E.* = 0.152, *p* = 0.029), but not with search or actual consumption.

Conversely, exposure to messages emphasizing cost savings, sensory enjoyment, and the natural content of legume-based foods was not associated with any differences in the outcomes of interest compared to the control and showed no significant indirect effects on future consumption intention.

### 4.4. Moderated Serial Mediation Model

Results of the moderated serial mediation model are shown in the second and third columns (“Non-Intenders” and “Intenders”) of Table 3 and Table A4.

In general (i.e., among participants in the control group), intenders were significantly more likely than non-intenders to have searched for legume-based products (*B* = 0.931, *S.E*. = 0.197, *p* < 0.001), to have consumed them (*B* = 0.367, *S.E.* = 0.180, *p* = 0.041), and to have a greater intention to consume such products in the future (*B* = 0.992, *S.E.* = 0.185, *p* < 0.001). Indirect effects also showed that being an intender was positively associated with future intention through search (*B* = 0.801, *S.E.* = 0.176, *p* < 0.001) and through the full serial path (*B* = 0.104, *S.E.* = 0.030, *p* = 0.001), but not through consumption alone.

With regard to search behavior, moderation effects by intention status were observed for mood and health messages. Among non-intenders, reading mood (*B* = 0.639, *S.E.* = 0.226, *p* = 0.005; Wald test: *χ*^2^(1) = 5.046, *p* = 0.025) and health (*B* = 0.597, *S.E.* = 0.222, *p* = 0.007; Wald test: *χ*^2^(1) = 4.391, *p* = 0.036) messages was associated with a significantly higher likelihood of searching for legume-based foods compared to non-intenders in the control group, whereas no such effect was found among intenders.

As to actual consumption, moderation effects emerged for the sustainability condition. Among intenders, reading sustainability messages was associated with a higher likelihood of consuming legume-based foods (*B* = 0.472, *S.E.* = 0.202, *p* = 0.019; Wald test: *χ*^2^(1) = 4.020, *p* = 0.045) compared to intenders in the control group, while this effect was not observed for non-intenders.

As for the intention to consume legume-based foods in the future, no moderation effects were observed.

Furthermore, several moderation effects were observed in the analysis of the indirect effects. Compared to non-intenders in the control group, non-intenders who read mood and health messages showed higher intention through search (*Mood*: *B* = 0.550, *S.E.* = 0.196, *p* = 0.005; Wald test: *χ*^2^(1) = 4.952, *p* = 0.026; *Health*: *B* = 0.514, *S.E.* = 0.192, *p* = 0.008; Wald test: *χ*^2^(1) = 4.323, *p* = 0.038) and through the full serial path (*Mood*: *B* = 0.504, *S.E.* = 0.179, *p* = 0.005; Wald test: *χ*^2^(1) = 5.011, *p* = 0.025; *Health*: *B* = 0.471, *S.E.* = 0.176, *p* = 0.007; Wald test: *χ*^2^(1) = 4.381, *p* = 0.036). These effects, however, were not observed among intenders. Additionally, compared to non-intenders in the control group, non-intenders exposed to health (*B* = −0.093, *S.E.* = 0.033, *p* = 0.005; Wald test: *χ*^2^(1) = 5.240, *p* = 0.022), price (*B* = −0.075, *S.E.* = 0.032, *p* = 0.021; Wald test: *χ*^2^(1) = 4.942, *p* = 0.026), and sustainability (*B* = −0.068, *S.E.* = 0.032, *p* = 0.031; Wald test: *χ*^2^(1) = 6.221, *p* = 0.013) messages showed a decrease in intention via reduced consumption. Again, these effects were not found among intenders.

## 5. Discussion

This experiment tested whether recommendation messages grounded in different food choice motives promoted search, consumption, and intention to consume legume-enriched products in a representative sample of Italian adults. It also examined whether these associations varied as a function of participants’ initial motivation to replace animal foods with plant-based alternatives.

Preliminary analyses confirmed limited legume-enriched food consumption, consistent with European data [59]. Non-intenders were more likely to be men, older, and less educated—groups generally less open to plant-based alternatives and more resistant to dietary change e.g., [24,60]. As expected, they also reported lower awareness and consumption of legume-enriched products.

Results suggested that the dynamics associated with reading the messages differed depending on whether the analysis was conducted for the total sample or separately by participants’ initial motivation to reduce their consumption of animal foods.

In the overall sample, messages emphasizing mood, health, convenience, and sustainability were positively associated with recipients’ future intention to consume legume-enriched foods, though via distinct pathways. This pattern is consistent with participant ratings, which indicated that mood, health, and sustainability messages were generally better received and elicited higher levels of trust, involvement, and positive tone.

Mood messages were associated with higher levels of future intention only when the pathway involved higher product search without subsequent consumption; when the pathway also involved consumption, the indirect effect of message exposure on intention was no longer statistically significant. This suggests that actual consumption likely yielded heterogeneous experiences among participants, including negative sensory reactions (e.g., disappointment, disgust), consistent with the weaker path coefficient from consumption to intention compared to that from search to intention. As a result, mood-based messages may not sufficiently support intention formation in the face of potentially negative experiences and may be most effective in the exploration phase, before the product is tested.

Sustainability messages, by contrast, showed a positive serial effect on intention through both search and consumption, suggesting that sustainability is a robust motive to leverage. Unlike mood messages, which appeared to be more effective before consumption took place, reading sustainability messages remained positively associated with intention even after actual consumption. Although environmental motives are sometimes portrayed as peripheral or effective mainly when paired with personal motives e.g., [61,62,63], the present results indicate that sustainability appeals may be influential on their own, consistent with findings that environmental motives strongly drive organic food purchases [64].

Health and convenience messages were directly associated with higher intention but showed no significant associations with search or consumption behaviors, pointing to a superficial rather than behavioural engagement. Furthermore, price, natural content, and sensory appeal messages had no significant effects on intention, search, or consumption, suggesting that these motives may lack persuasive strength as standalone frames.

Moderation analyses showed that, among individuals who did not perceive any reason to reduce their consumption of animal-based foods (i.e., non-intenders), mood and health messages were associated with higher intention only when an initial search phase occurred, supporting evidence that personal motives (e.g., health, satiety) tend to be stronger drivers of food choice than altruistic motives (e.g., animal or environmental welfare) among individuals in the earlier stages of behavioral change [47]. When the pathway included consumption but not prior search, exposure to health messages was negatively associated with future intention; a similar pattern emerged for price and sustainability messages, suggesting a boomerang effect if these individuals are pushed prematurely into action. These results underscore the importance of stimulating an initial exploratory phase in people not yet motivated to change their diet. Search behaviour may offer a low-threshold, psychologically acceptable form of engagement in early change stages. Unlike actual consumption, which requires more effort, commitment, and openness to change, searching for information or products can feel safer and less demanding. If this preliminary phase is bypassed, a boomerang effect can occur, where exposure to the message is followed by lower consumption compared to non-intenders in the control group.

Among individuals already motivated to reduce animal-based food consumption (i.e., intenders), only sustainability messages were associated with increased consumption, whereas no significant associations emerged for search or future intention. This likely reflects these individuals’ greater familiarity with legume-enriched foods (e.g., where to find them) and pre-existing consumption intention. In this context, sustainability messages likely reinforced the behaviours that intenders were already motivated to engage in, demonstrating the power of value-congruent messages in maintaining identity-conforming actions, in line with self-determination theory [65].

Overall, mood, health, and especially sustainability emerged as the most promising content to promote acceptance and consumption of legume-enriched foods, even when these products are presented as direct alternatives to animal foods rather than unique options [66]. The present findings extend prior evidence on meat and legume consumption e.g., [27,28,29] by showing that ego-oriented motives (i.e., mood and health) may be particularly effective in arousing initial curiosity among individuals not yet motivated to reduce their consumption of animal foods, whereas altruistic motives (i.e., sustainability) appear more appropriate for individuals already committed to such changes. Tailoring persuasive strategies to motivational orientation or stage of change is therefore warranted [67]. However, the observed divergence between intention and behaviour highlights the limits of current theoretical models and underscores the need for further research to replicate and elaborate on these insights.

### Study Limitations and Future Directions

Despite the experimental design and the inclusion of multiple outcome variables along the behaviour change continuum, several limitations should be acknowledged. First, a set of limitations relates to measurement and design choices. Although participants who never read the messages were excluded, exposure did not guarantee actual attention or cognitive processing. In addition, outcomes were assessed shortly after message exposure, capturing only short-term effects. Longer follow-up assessments are therefore needed to examine whether initial curiosity or intention translates into sustained behaviour, particularly among less motivated individuals. Moreover, the use of self-reported measures may have led participants to overreport sustainable behaviors and intentions due to social desirability concerns. Finally, although widely used in psychological research, single-item and ordinal measures may have introduced some degree of measurement bias. Similarly, the dichotomization of search and consumption outcomes, adopted to capture whether participants crossed a meaningful behavioral threshold under constrained temporal conditions, may have resulted in a loss of information compared to the use of full ordinal scales.

Second, limitations related to individual differences and motivational readiness should be considered. The classification of participants into “intenders” and “non-intenders” relied on a single item and implied the aggregation of multiple motivational profiles within the same group. As a consequence, moderation effects should be interpreted with caution, as this categorization represents a simplified proxy of individuals’ stage of change. Future studies could adopt validated multi-item measures from stage-based models to more precisely capture readiness for dietary change. In addition, the study did not explicitly assess participants’ responsibility for food purchasing or preparation within the household, which may influence how intentions translate into actual behaviour in domestic contexts. Future research could therefore examine the moderating role of food-related decision-making responsibilities in shaping the intention–behaviour relationship.

Third, contextual and communication-related boundaries should be acknowledged. The study was conducted exclusively in Italy; cultural differences in dietary habits, familiarity with legumes, and market availability of legume-enriched products may therefore limit the generalizability of the findings to other national contexts. Moreover, the messages were intentionally based on single motives to facilitate comparisons across conditions, but this approach oversimplifies real-world food communication, which often combines multiple arguments. Future research should investigate whether combining motivational appeals enhances message persuasiveness, particularly among less motivated target groups. Previous studies suggest, for instance, that environmental claims may be more effective when coupled with self-serving motives [61,68,69], and that multifaceted framing can increase persuasiveness [70].

## 6. Conclusions

This study suggests that recommendation messages grounded in different food choice motives may play distinct roles in shaping consumers’ engagement with legume-enriched foods across the behavior change pathway. Rather than exerting a uniform persuasive effect, motivational appeals appear to be differentially associated with exploratory behaviors, experiential engagement, and the consolidation of future intentions.

Across conditions, messages appealing to sustainability, health, and mood were associated with future intentions to consume legume-enriched foods, although their patterns of influence differed. Sustainability messages were associated with future intention both following initial exploration and after actual consumption, suggesting that such appeals may be relatively robust to variations in product experience and may support longer-term engagement. In contrast, mood-based messages were primarily associated with increased curiosity when linked to product search without subsequent consumption, indicating that these appeals may be more sensitive to heterogeneous or less favorable product experiences.

Differences also emerged as a function of participants’ baseline motivation to reduce animal-based food consumption. Among non-intenders, health- and mood-focused messages were associated with higher future intention mainly through pathways involving product search, suggesting that more self-focused motives may serve as accessible entry points at earlier stages of dietary change. Among intenders, instead, sustainability-based messages were the only ones associated with higher consumption, potentially reflecting the reinforcement of existing value-aligned motivations rather than the initiation of new interest.

Taken together, these findings underscore the importance of aligning message framing with consumers’ stage of engagement. While appeals to personal benefits such as health or mood may help initiate interest among less engaged consumers, sustainability-oriented messages may be more effective in supporting the consolidation of intentions and behaviors among individuals already inclined toward plant-based eating. From a practical perspective, communication strategies aimed at promoting legume-enriched foods may therefore benefit from a staged approach, in which different motivational framings are strategically deployed depending on the targeted phase of behavior change

## Figures and Tables

**Table 1:** Constructs and Items Measured in the Survey Instrument.

Time	Construct	α	Example Item	*N* of Items	Response Scale
1	Awareness of legume-enriched foods	-	“Have you ever heard of legume-enriched foods?”	1	1 = Yes 2 = No 3 = I don’t know
1	Prior consumption of legume-enriched foods	-	“How often have you eaten legume-enriched foods in the last month?”	1	1 = Never 2 = Less than once a week 3 = 1–3 times a week 4 = 4–6 times a week 5 = Once or more a day
1	Intention to replace animal foods with plant-based alternatives	-	“Have you replaced animal foods (such as meat, fish, eggs, and dairy products) with plant-based alternatives (such as plant-based burgers, cereals, legumes, and plant-based milk) in the last few years?”	1	1 = No, I don’t see the point 2 = No, but I plan to do it soon, although I don’t know exactly how 3 = No, but I have already considered it, although I haven’t changed my consumption of animal foods 4 = No, but I am considering it soon and know how 5 = No, because I don’t eat animal foods anyway 6 = No, it’s for another reason 7 = Yes, for less than two years 8 = Yes, for more than two years
2	Intention to consume legume-enriched foods	0.95	“In the next week, I plan to consume legume-enriched foods.”	3	Likert scale 1 = Completely disagree 7 = Completely agree
2	Search for legume-enriched foods	-	“To what extent have you searched for or tried to find legume-enriched foods in the past week?”	1	Likert-type scale 1 = Not at all 2 = A little 3 = Somewhat 4 = Quite a lot 5 = Very much
2	Consumption of legume-enriched foods	-	“How often have you eaten legume-enriched foods in the past week?”	1	Likert-type scale 1 = Never 2 = Once 3 = 2–3 times 4 = 4–5 times 5 = 6 or more times
2	Frequency of message reading	-	“How often have you read the messages?”	1	Likert-type scale 1 = Never to 4 = Always
2	Perceived tone of the message	-	“Overall, how would you rate the tone of the information in the messages you read?”	1	Semantic differential scale 1 = Extremely negative 7 = Extremely positive
2	Message involvement	0.89	“The messages I read made me think.”	3	Likert scale 1 = Completely disagree 7 = Completely agree
2	Message trust	0.95	“Do you believe the information is truthful?”	3	Likert scale 1 = Not at all 7 = Extremely
2	Systematic processing	0.84	“As I read the messages, I thought about what action I could take based on what I had read.”	5	Likert scale 1 = Completely disagree 7 = Completely agree
2	Perceived threat to freedom	0.90	“The messages I received tried to limit my freedom of choice.”	4	Likert scale 1 = Completely disagree 7 = Completely agree
2	Negative emotions elicited by the messages (anger, fear, and anxiety)	0.95	“To what extent reading the messages made you feel…annoyed.”	9	Likert-type scale 1 = Not at all 5 = Completely
2	Positive emotions elicited by the messages (hope and satisfaction)	0.89	“To what extent reading the messages made you feel… optimistic.”	6	Likert-type scale 1 = Not at all 5 = Completely

**Table 2:** Descriptive Statistics of Study Variables (Time 1 and Time 2) and Message Evaluation Variables.

Condition										
	Overall *N* = 1361	Health *n* = 168	Price *n* = 158	Sensory Appeal *n* = 167	Natural Content *n* = 159	Convenience *n* = 169	Sustainability *n* = 161	Mood *n* = 171	Control *n* = 208	*χ* ^2^
**Variables Measured at T1**										
Intention to Replace Animal Foods										
Non-intender	42.4%	41.7%	46.8%	44.9%	42.1%	37.9%	42.9%	37.4%	45.2%	*χ*^2^(7) = 5.57, *p* = 0.590
Intender	57.6%	58.3%	53.2%	55.1%	57.9%	62.1%	57.1%	62.6%	54.8%	
Awareness of Legume-Enriched Foods										
Unaware	21.4%	19.6%	23.4%	19.8%	23.9%	21.9%	18.6%	22.2%	21.6%	*χ*^2^(7) = 2.38, *p* = 0.936
Aware	78.6%	80.4%	76.6%	80.2%	76.1%	78.1%	81.4%	77.8%	78.4%	
Prior Consumption of Legume-Enriched Foods										
Never	41.1%	39.3%	44.3%	43.7%	43.4%	36.7%	36.6%	40.9%	43.8%	*χ*^2^(28) = 28.19, *p* = 0.454
Less than once a week	36.6%	35.1%	33.5%	38.3%	39.6%	39.6%	34.2%	36.3%	36.1%	
1–3 times a week	20%	23.2%	22.2%	16.8%	15.1%	21.9%	24.2%	19.9%	17.3%	
4–6 times a week	2.1%	1.8%	0.0%	1.2%	1.9%	1.2%	5.0%	2.3%	2.9%	
Once or more per day	0.2%	0.6%	0.0%	0.0%	0.0%	0.6%	0.0%	0.6%	0.0%	
**Variables Measured at T2**										
Intention to Consume	4.65 (1.45)	4.77 (1.36)	4.56 (1.49)	4.44 (1.52)	4.58 (1.37)	4.80 (1.35)	4.88 (1.50)	4.78 (1.45)	4.44 (1.52)	-
Search (1 = Yes)	36.3%	40.5%	32.9%	32.3%	30.2%	39.1%	43.5%	41.5%	31.7%	-
Consumption (1 = Yes)	62.7%	62.5%	60.8%	56.3%	56.0%	63.9%	71.4%	64.9%	64.9%	-
Message Tone	5.64 (1.21)	5.74 (1.20)	5.46 (1.26)	5.46 (1.25)	5.50 (1.26)	5.67 (1.18)	5.73 (1.20)	5.88 (1.10)	-	-
Message Involvement	5.02 (1.26)	5.20 (1.18)	4.93 (1.34)	4.78 (1.36)	4.83 (1.32)	5.04 (1.10)	5.32 (1.17)	5.03 (1.27)	-	-
Message Trust	4.60 (1.13)	4.81 (1.13)	4.42 (1.14)	4.47 (1.15)	4.41 (1.17)	4.67 (0.99)	4.83 (1.25)	4.60 (1.02)	-	-
Systematic Processing	4.75 (1.07)	4.84 (1.11)	4.68 (1.07)	4.57 (1.17)	4.63 (1.06)	4.81 (0.95)	4.94 (0.96)	4.78 (1.13)	-	-
Threat to Freedom	2.45 (1.39)	2.35 (1.34)	2.47 (1.37)	2.49 (1.44)	2.72 (1.58)	2.44 (1.42)	2.42 (1.42)	2.27 (1.15)	-	-
Message-induced Negative Emotions	1.20 (0.44)	1.27 (0.55)	1.19 (0.42)	1.19 (0.48)	1.23 (0.50)	1.17 (0.36)	1.21 (0.42)	1.12 (0.31)	-	-
Message-induced Positive Emotions	3.08 (0.85)	3.14 (0.85)	3.02 (0.83)	3.02 (0.83)	2.90 (0.89)	3.16 (0.76)	3.16 (0.84)	3.15 (0.91)	-	-

Note: Means and standard deviations are reported for continuous variables; percentages are reported for dichotomous variables. Chi-square statistics are reported only for variables measured at Time 1 to assess the success of randomization.

**Table 3:** Direct Effects from the Estimated Serial Mediation (“All”) and Moderated Serial Mediation (“Non-Intenders” and “Intenders”) Models.

All					Non Intenders				Intenders				
Condition	Est.	*S.E.*	*p*	95% CI	Est.	*S.E.*	*p*	95% CI	Est.	*S.E.*	*p*	95% CI	Wald Test
***Direct Effects on Search***													
Mood	**0.261**	**0.132**	**0.049**	**[0.001–0.521]**	**0.639**	**0.226**	**0.005**	**[0.196–1.082]**	0.005	0.169	0.979	[−0.327–0.336]	***χ*^2^(1) = 5.046; *p* = 0.025**
Health	0.234	0.133	0.079	[−0.027–0.495]	**0.597**	**0.222**	**0.007**	**[0.162–1.032]**	0.008	0.173	0.965	[−0.332–0.347]	***χ*^2^(1) = 4.391; *p* = 0.036**
Price	0.033	0.137	0.811	[−0.237–0.302]	0.388	0.223	0.083	[−0.050–0.825]	−0.162	0.182	0.374	[−0.518–0.195]	-
Sensory Appeal	0.017	0.136	0.901	[−0.249–0.283]	0.291	0.225	0.197	[−0.151–0.733]	−0.138	0.177	0.437	[−0.484–0.209]	-
Natural Content	−0.044	0.138	0.752	[−0.315–0.227]	0.331	0.231	0.152	[−0.122–0.783]	−0.281	0.179	0.116	[−0.631–0.069]	-
Convenience	0.197	0.133	0.139	[−0.064–0.459]	0.316	0.234	0.177	[−0.143–0.776]	0.074	0.170	0.661	[−0.258–0.407]	-
Sustainability	**0.311**	**0.134**	**0.021**	**[0.048–0.574]**	**0.487**	**0.225**	**0.030**	**[0.047–0.928]**	0.219	0.176	0.213	[−0.126–0.565]	*χ*^2^(1) = 0.880; *p* = 0.348
Intender					**Est. = 0.931**		***S.E.* = 0.197**		***p* = 0.000**		**95% CI = [0.544–1.317]**		
***Direct Effects on Consumption***													
Mood	−0.208	0.125	0.097	[−0.454–0.038]	**−0.534**	**0.205**	**0.009**	**[−0.936–−0.133]**	−0.031	0.164	0.848	[−0.354–0.291]	*χ*^2^(1) = 3.662; *p* = 0.056
Health	**−0.251**	**0.126**	**0.046**	**[−0.498–−0.004]**	**−0.659**	**0.187**	**<0.001**	**[−1.026–−0.291]**	0.005	0.178	0.978	[−0.344–0.354]	***χ*^2^(1) = 6.623; *p* = 0.010**
Price	−0.136	0.127	0.284	[−0.385–0.113]	**−0.527**	**0.195**	**0.007**	**[−0.910–−0.145]**	0.139	0.178	0.438	[−0.211–0.488]	***χ*^2^(1) = 6.330; *p* = 0.012**
Sensory Appeal	−0.238	0.123	0.054	[−0.480–0.004]	−0.367	0.209	0.079	[−0.777–0.042]	−0.199	0.156	0.203	[−0.505–0.107]	-
Natural Content	−0.198	0.123	0.110	[−0.439–0.044]	−0.392	0.208	0.059	[−0.799–0.015]	−0.114	0.158	0.471	[−0.423–0.195]	-
Convenience	−0.184	0.127	0.147	[−0.433–0.064]	**−0.437**	**0.209**	**0.036**	**[−0.847–−0.028]**	−0.019	0.170	0.909	[−0.352–0.313]	*χ*^2^(1) = 2.417; *p* = 0.120
Sustainability	−0.065	0.129	0.614	[−0.317–0.187]	**−0.481**	**0.199**	**0.016**	**[−0.871–−0.091]**	0.299	0.190	0.115	[−0.073–0.672]	***χ*^2^(1) = 8.066; *p* = 0.005**
Search	**0.798**	**0.024**	**<0.001**	**[0.751–0.844]**	**Est. = 0.788**		***S.E.* = 0.025**		***p* = 0.000**		**95% CI = [0.740–0.836]**		
Intender					**Est. = −0.367**		***S.E.* = 0.187**		***p* = 0.050**		**95% CI = [−0.733–−0.000]**		
***Direct Effects on Intention***													
Mood	0.094	0.135	0.489	[−0.171–0.358]	0.000	0.213	1.000	[−0.418–0.418]	0.089	0.171	0.601	[−0.246–0.424]	-
Health	0.124	0.139	0.373	[−0.149–0.397]	−0.004	0.218	0.987	[−0.431–0.424]	0.157	0.179	0.379	[−0.193–0.508]	-
Price	0.103	0.142	0.467	[−0.176–0.382]	−0.055	0.207	0.789	[−0.461–0.350]	0.173	0.198	0.380	[−0.214–0.561]	-
Sensory Appeal	0.013	0.128	0.920	[−0.239–0.264]	0.010	0.204	0.963	[−0.391–0.410]	−0.046	0.163	0.779	[−0.366–0.274]	-
Natural Content	0.214	0.139	0.123	[−0.058–0.486]	−0.114	0.207	0.584	[−0.520–0.293]	0.368	0.190	0.052	[−0.004–0.740]	-
Convenience	0.184	0.134	0.171	[−0.080–0.447]	0.154	0.210	0.464	[−0.258–0.566]	0.156	0.181	0.390	[−0.199–0.511]	-
Sustainability	0.125	0.132	0.344	[−0.134–0.384]	−0.129	0.208	0.536	[−0.537–0.280]	0.268	0.186	0.150	[−0.097–0.633]	-
Search	**0.933**	**0.049**	**<0.001**	**[0.837–1.028]**	**Est. = 0.861**		***S.E.* = 0.048**		***p* = 0.000**		**95% CI = [0.768–0.954]**		
Consumption	**0.143**	**0.033**	**<0.001**	**[0.079–0.208]**	**Est.= 0.142**		***S.E.* = 0.033**		***p* = 0.000**		**95% CI = [0.078–0.206]**		-
Intender					Est.= 0.138		*S.E*. = 0.198		*p* = 0.485		95% CI = [−0.250–0.527]		-

Note: Unstandardized estimates, standard errors, and 95% confidence intervals are reported. Wald tests were computed only when at least one effect (for intenders or non-intenders) was significantly different from zero. Results that reached statistical significance (*p* ≤ 0.05) are shown in bold.

## Data Availability

The de-identified individual participant data supporting the findings of this study, together with the data dictionary, are publicly available on the Open Science Framework (OSF) repository at https://osf.io/ft3g7.

## Appendix A

**Table A1:** Comparison Between the Original Representative Sample and the Final Analytical Sample.

	Original Representative Sample		Final Sample Used in the Analysis	
	*N*	%	*N*	%
**Gender**				
Man	957	44.3%	571	42%
Woman	1202	55.6%	789	58%
Other	1	0.0%	1	0.1%
**Age**				
18–24	137	6.3%	68	5%
25–34	225	10.4%	131	9.6%
35–44	303	14.0%	174	12.8%
45–54	467	21.6%	307	22.6%
55–64	476	22.0%	318	23.4%
>64	552	25.6%	363	26.7%
**Geographical Provenience**				
Northwest	606	28.1%	408	30.0%
Northeast	433	20.0%	271	19.9%
Center	427	19.8%	291	21.4%
South	467	21.6%	265	19.5%
Islands	227	10.5%	126	9.3%
**Education**				
No university degree	1377	63.7%	856	62.9%
University degree	780	36.1%	505	37.1%

**Table A2:** Descriptive Statistics for Participants’ Socio-Demographic Characteristics and Dietary Habits.

Variables			Condition							
	Overall *N* = 1361	Health *n* = 168	Price *n* = 158	Sensory Appeal *n* = 167	Natural Content *n* = 159	Convenience *n* = 169	Sustainability *n* = 161	Mood *n* = 171	Control *n* = 208	*χ* ^2^
**Gender**										
Man	42%	39.3%	46.2%	44.3%	39.6%	35.5%	36.6%	46.2%	46.6%	*χ*^2^(14) = 16.00, *p* = 0.313
Woman	58%	60.7%	53.8%	55.7%	60.4%	64.5%	63.4%	53.8%	52.9%	
Other	0.1%	0.0%	0.0%	0.0%	0.0%	0.0%	0.0%	0.0%	0.5%	
**Age**										
18–24	5%	2.4%	6.3%	4.8%	3.8%	1.2%	8.1%	5.8%	7.2%	*χ*^2^(35) = 48.71, *p* = 0.062
25–34	9.6%	11.3%	9.5%	7.8%	10.1%	8.9%	9.3%	7.0%	12.5%	
35–44	12.8%	17.3%	12.7%	9.6%	10.1%	12.4%	8.7%	17.0%	13.9%	
45–54	22.6%	22.6%	17.1%	31.1%	23.9%	27.8%	21.7%	17.0%	19.7%	
55–64	23.4%	21.4%	20.9%	24.0%	22.0%	21.9%	26.7%	25.7%	24.0%	
>64	26.7%	25.0%	33.5%	22.8%	30.2%	27.8%	25.5%	27.5%	22.6%	
**Geographical Provenience**										
Northwest	30%	33.9%	31.0%	30.5%	29.6%	29.0%	28.6%	24.0%	32.7%	*χ*^2^(28) = 25.09, *p* = 0.623
Northeast	19.9%	24.4%	22.2%	22.8%	19.5%	15.4%	19.9%	21.1%	15.4%	
Center	21.4%	17.9%	20.3%	18.0%	22.6%	25.4%	18.6%	22.8%	24.5%	
South	19.5%	16.1%	16.5%	19.2%	18.2%	18.3%	26.1%	22.2%	19.2%	
Islands	9.3%	7.7%	10.1%	9.6%	10.1%	11.8%	6.8%	9.9%	8.2%	
**Education**										
No university degree	62.9%	59.5%	56.3%	62.3%	69.2%	61.5%	68.9%	65.5%	60.6%	*χ*^2^(7) = 10.09, *p* = 0.184
University degree	37.1%	40.5%	43.7%	37.7%	30.8%	38.5%	31.1%	34.5%	39.4%	
**Employment Status**										
Employed	56.1%	56.5%	48.7%	56.9%	51.6%	57.4%	59.6%	59.6%	57.2%	*χ*^2^(28) = 24.24, *p* = 0.668
Unemployed	4.8%	4.2%	4.4%	6.6%	6.9%	4.1%	3.1%	7%	2.4%	
Retired	21.2%	20.8%	26.6%	18%	23.9%	21.9%	18%	19.3%	21.2%	
Student	6%	5.4%	7.6%	4.8%	5.7%	3.6%	8.7%	4.7%	7.7%	
Other	12%	13.1%	12.7%	13.8%	11.9%	13%	10.6%	9.4%	11.5%	
**Diet**										
Omnivorous	82.5%	86.3%	80.4%	80.2%	81.8%	85.2%	80.7%	81.3%	83.7%	*χ*^2^(35) = 34.20, *p* = 0.506
Vegetarian	3.7%	1.8%	5.1%	4.8%	3.1%	3.6%	5.0%	2.9%	3.8%	
Vegan	1.3%	1.8%	1.9%	0.0%	1.3%	0.0%	3.1%	0.6%	1.9%	
Pescetarian	2.3%	2.4%	4.4%	2.4%	1.3%	1.2%	3.1%	2.9%	1.0%	
Flexitarian	8.6%	6.0%	7.0%	11.4%	8.8%	9.5%	7.5%	10.5%	8.2%	
Other	1.5%	1.8%	1.3%	1.2%	3.8%	0.6%	0.6%	1.8%	1.4%	

**Table A3:** Correlations Among Study Variables (Time 2) and Message Evaluation Variables.

Variables	1.	2.	3.	4.	5.	6.	7.	8.	9.
1. Intention to Consume	-								
2. Search (1 = *Yes*)	0.536 ***	-							
3. Consumption (1 = *Yes*)	0.550 ***	0.498 ***	-						
4. Message Tone	0.535 ***	0.314 ***	0.260 ***	-					
5. Message Involvement	0.658 ***	0.431 ***	0.376 ***	0.669 ***	-				
6. Message Trust	0.576 ***	0.428 ***	0.329 ***	0.655 ***	0.650 ***	-			
7. Systematic Processing	0.603 ***	0.423 ***	0.376 ***	0.548 ***	0.725 ***	0.566 ***	-		
8. Threat to Freedom	−0.343 ***	−0.101 ***	−0.131 ***	−0.447 ***	−0.362 ***	−0.388 ***	−0.246 ***	-	
9. Negative Emotions	−0.155 ***	0.061 *	0.004	−0.320 ***	−0.202 ***	−0.189 ***	−0.126 ***	0.428 ***	-
10. Positive Emotions	0.519 ***	0.433 ***	0.314 ***	0.601 ***	0.652 ***	0.637 ***	0.585 ***	−0.300 ***	−0.131 ***

Note: Pearson correlations are reported for associations between continuous variables, point-biserial correlations for associations between continuous and dichotomous variables, and phi coefficients for associations between dichotomous variables. *** *p* < 0.001, * *p* < 0.05.

**Table A4:** Indirect and Total Effects from the Estimated Serial Mediation (“All”) and Moderated Serial Mediation (“Non-Intenders” and “Intenders”) Models.

	All				Non–Intenders				Intenders				Wald Test
	Est.	*S.E.*	*p*	95% CI	Est.	*S.E.*	*p*	95% CI	Est.	*S.E.*	*p*	95% CI	
***Indirect Effects Intervention → Search → Intention***													
Mood	**0.243**	**0.123**	**0.048**	**[0.002–0.485]**	**0.550**	**0.196**	**0.005**	**[0.166–0.934]**	0.004	0.146	0.979	[−0.281–0.289]	***χ*^2^(1) = 4.952; *p* = 0.026**
Health	0.218	0.124	0.078	[−0.025–0.462]	**0.514**	**0.192**	**0.008**	**[0.137–0.891]**	0.007	0.149	0.965	[−0.285–0.299]	***χ*^2^(1) = 4.323; *p* = 0.038**
Price	0.031	0.128	0.811	[−0.220–0.282]	0.334	0.192	0.083	[−0.043–0.711]	−0.139	0.157	0.375	[−0.447–0.168]	-
Sensory Appeal	0.016	0.126	0.901	[−0.232–0.263]	0.251	0.194	0.197	[−0.130–0.631]	−0.118	0.153	0.438	[−0.418–0.181]	-
Natural Content	−0.041	0.129	0.752	[−0.294–0.212]	0.285	0.199	0.152	[−0.105–0.675]	−0.242	0.155	0.119	[−0.546–0.063]	-
Convenience	0.184	0.124	0.137	[−0.059–0.427]	0.272	0.202	0.176	[−0.123–0.667]	0.064	0.146	0.661	[−0.222–0.350]	-
Sustainability	**0.290**	**0.125**	**0.020**	**[0.045–0.535]**	**0.419**	**0.194**	**0.031**	**[0.038–0.800]**	0.189	0.151	0.211	[−0.107–0.485]	*χ*^2^(1) = 0.874; *p* = 0.350
Intender					**Estimate = 0.801**		***S.E.* = 0.176**		***p* = 0.000**		**95% CI = [0.457–1.145]**		*-*
***Indirect Effects Intervention → Consumption → Intention***													
Mood	−0.030	0.020	0.131	[−0.069–0.009]	**−0.076**	**0.033**	**0.024**	**[−0.141––0.010]**	−0.004	0.023	0.849	[−0.050–0.042]	*χ*^2^(1) = 3.302; *p* = 0.069
Health	−0.036	0.020	0.076	[−0.076–0.004]	**−0.093**	**0.033**	**0.005**	**[−0.159–−0.028]**	0.001	0.025	0.978	[−0.049–0.050]	***χ*^2^(1) = 5.240; *p* = 0.022**
Price	−0.020	0.019	0.306	[−0.057–0.018]	**−0.075**	**0.032**	**0.021**	**[−0.138–−0.011]**	0.020	0.025	0.440	[−0.030–0.070]	***χ*^2^(1) = 4.942; *p* = 0.026**
Sensory Appeal	−0.034	0.020	0.088	[−0.073–0.005]	−0.052	0.032	0.098	[−0.114–0.010]	−0.028	0.024	0.236	[−0.075–0.018]	-
Natural Content	−0.028	0.020	0.151	[−0.067–0.010]	−0.056	0.032	0.082	[−0.118–0.007]	−0.016	0.023	0.487	[−0.062–0.029]	-
Convenience	−0.026	0.020	0.185	[−0.066–0.013]	−0.062	0.033	0.063	[−0.127–0.003]	−0.003	0.024	0.910	[−0.050–0.045]	-
Sustainability	−0.009	0.019	0.619	[−0.046–0.027]	**−0.068**	**0.032**	**0.031**	**[−0.130–−0.006]**	0.042	0.028	0.127	[−0.012–0.097]	***χ*^2^(1) = 6.221; *p* = 0.013**
Intender					Estimate = −0.052		*S.E.* = 0.027		*p =* 0.056		95% CI = [−0.105–0.001]		***-***
***Indirect Effects Intervention → Search → Consumption → Intention***													
Mood	0.030	0.017	0.077	[−0.003–0.063]	**0.504**	**0.179**	**0.005**	**[0.152–0.855]**	0.004	0.133	0.979	[−0.258–0.265]	***χ*^2^(1) = 5.011; *p* = 0.025**
Health	0.027	0.017	0.106	[−0.006–0.059]	**0.471**	**0.176**	**0.007**	**[0.127–0.815]**	0.006	0.136	0.965	[−0.261–0.274]	***χ*^2^(1) = 4.381; *p* = 0.036**
Price	0.004	0.016	0.812	[−0.027–0.035]	0.306	0.177	0.083	[−0.040–0.652]	−0.128	0.143	0.374	[−0.409–0.154]	-
Sensory appeal	0.002	0.016	0.901	[−0.029–0.032]	0.230	0.178	0.198	[−0.120–0.579]	−0.108	0.140	0.437	[−0.382–0.165]	-
Natural Content	−0.005	0.016	0.751	[−0.036–0.026]	0.261	0.182	0.153	[−0.097–0.618]	−0.222	0.141	0.116	[−0.498–0.055]	-
Convenience	0.023	0.016	0.170	[−0.010–0.055]	0.250	0.185	0.177	[−0.113–0.612]	0.059	0.134	0.662	[−0.204–0.321]	-
Sustainability	**0.036**	**0.018**	**0.044**	**[0.001–0.070]**	**0.384**	**0.178**	**0.031**	**[0.036–0.733]**	0.173	0.139	0.213	[−0.099–0.445]	*χ*^2^(1) = 0.878; *p* = 0.349
Intender	**-**				**Estimate = 0.104**		***S.E.* = 0.030**		***p* = 0.001**		**95% CI = [0.044–0.164]**		*-*
***Total Effects on Consumption***													
Mood	0.000	0.133	0.999	[−0.260–0.261]	−0.030	0.204	0.881	[−0.431–0.370]	−0.028	0.178	0.876	[−0.377–0.321]	-
Health	−0.064	0.133	0.630	[−0.325–0.197]	−0.188	0.198	0.344	[−0.577–0.201]	0.011	0.183	0.952	[−0.348–0.370]	-
Price	−0.110	0.135	0.416	[−0.374–0.155]	−0.222	0.195	0.257	[−0.605–0.161]	0.011	0.191	0.954	[−0.363–0.385]	-
Sensory appeal	−0.224	0.132	0.089	[−0.484–0.035]	−0.138	0.195	0.480	[−0.519–0.244]	−0.307	0.181	0.090	[−0.663–0.048]	-
Natural Content	−0.232	0.134	0.083	[−0.495–0.030]	−0.132	0.201	0.513	[−0.526–0.262]	−0.335	0.181	0.064	[−0.690–0.020]	-
Convenience	−0.027	0.133	0.840	[−0.288–0.234]	−0.188	0.204	0.357	[−0.587–0.212]	0.039	0.180	0.827	[−0.314–0.392]	-
Sustainability	0.183	0.138	0.183	[−0.087–0.453]	−0.097	0.199	0.627	[−0.488–0.294]	**0.472**	**0.202**	**0.019**	**[0.077–0.868]**	***χ*^2^(1) = 4.020; *p* = 0.045**
Intender	-				**Estimate = 0.367**		***S.E.* = 0.180**		***p* = 0.041**		**95% CI = [0.015–0.720]**		***-***
***Total Effects on Intention***													
Mood	**0.337**	**0.146**	**0.021**	**[0.051–0.623]**	**0.546**	**0.215**	**0.011**	**[0.125–0.966]**	0.089	0.183	0.625	[−0.269–0.448]	*χ*^2^(1) = 2.616; *p* = 0.106
Health	**0.333**	**0.152**	**0.029**	**[0.035–0.631]**	**0.484**	**0.209**	**0.021**	**[0.074–0.894]**	0.166	0.202	0.412	[−0.230–0.561]	*χ*^2^(1) = 1.198; *p* = 0.274
Price	0.118	0.147	0.422	[−0.170–0.407]	0.247	0.195	0.204	[−0.134–0.628]	0.036	0.206	0.862	[−0.368–0.439]	-
Sensory appeal	−0.004	0.143	0.980	[−0.284–0.277]	0.241	0.206	0.243	[−0.164–0.645]	−0.208	0.182	0.253	[−0.564–0.149]	-
Natural Content	0.140	0.154	0.365	[−0.163–0.443]	0.153	0.205	0.457	[−0.249–0.554]	0.079	0.222	0.724	[−0.357–0.514]	-
Convenience	**0.364**	**0.152**	**0.017**	**[0.065–0.663]**	0.400	0.208	0.055	[−0.008–0.807]	0.225	0.207	0.277	[−0.181–0.632]	*χ*^2^(1) = 0.353; *p* = 0.553
Sustainability	**0.441**	**0.146**	**0.002**	**[0.156–0.727]**	0.277	0.198	0.163	[−0.112–0.666]	0.524	0.210	0.013	[0.112–0.936]	*χ*^2^(1) = 0.732; *p* = 0.392
Intender	**-**				**Estimate = 0.992**		***S.E.* = 0.185**		***p* = 0.000**		**95% CI = [0.629–1.355]**		*-*

Note: Unstandardized estimates, standard errors, and 95% confidence intervals are reported. Wald tests were computed only when at least one effect (for intenders or non-intenders) was significantly different from zero. Results that reached statistical significance (*p* ≤ 0.05) are shown in bold.
